# Self-selection of Asylum Seekers: Evidence From Germany

**DOI:** 10.1007/s13524-020-00873-9

**Published:** 2020-05-04

**Authors:** Lucas Guichard

**Affiliations:** 1 Institute for Employment Research, Nuremberg, Germany; 2 CERDI, CNRS, Université Clermont Auvergne, Clermont-Ferrand, France

**Keywords:** Refugee, Selection, Education, Individual-level data

## Abstract

I examine the pattern of selection on education of asylum seekers recently arrived in Germany from five key source countries: Afghanistan, Albania, Iraq, Serbia, and Syria. The analysis relies on original individual-level data collected in Germany combined with surveys conducted at origin. The results reveal a positive pattern of selection on education for asylum seekers who were able to flee Iraq and Syria, and the selection is neutral for individuals seeking asylum from Afghanistan and negative for asylum seekers from Albania and Serbia. I provide an interpretation of these patterns based on differences in the expected length of stay at destination, the migration costs faced by asylum seekers to reach Germany, and the size of migration networks at destination.

**Electronic supplementary material:**

The online version of this article (10.1007/s13524-020-00873-9) contains supplementary material, which is available to authorized users.

## Introduction

European countries experienced a short-lived surge in the arrival of asylum seekers from 2014 to 2016. More than 1.2 million first-time asylum applications were registered in the European Union in 2015 (Eurostat 2016), with Germany receiving approximately three-quarters of the applications lodged that year (BMI 2017).^1^ Because of the ongoing crisis in Syria, most asylum seekers came from there (41.5%), but some originated from other conflict-affected areas (18.1% from Afghanistan and Iraq combined) and from eastern European countries (5.9%). The size, diversity, and potential consequences of the large number of asylum seekers make it important to identify the characteristics of the newcomers, which are likely to affect the socioeconomic outcomes of the stayers in the origin country and of the natives at destination.^2^

The push factors behind the decisions of asylum seekers to migrate have been emphasized in the public debate as a pivotal feature differentiating them from economic migrants. The latter are often assumed to be able to choose whether to migrate, whereas asylum seekers are, in principle, forced to flee their country of origin because of threats to their lives. The drivers of economic migration have been widely studied in the literature. However, the determinants that explain who is able to leave the home country to seek asylum abroad have been rarely explored. In this study, I exploit individual-level and representative data related to the recent surge in asylum applications to Germany to improve the current limited knowledge and understanding about the mechanisms fostering the migration decision in the context of forced migration.

This study focuses on the self-selection on education of asylum seekers who arrived in Germany from 2013 or later.^3^ It delivers the first insights on this question for individuals drawn from the origin population of five source countries: Afghanistan, Albania, Iraq, Serbia, and Syria. These countries represent 65% of all first-time asylum applications lodged in Germany, and they offer an interesting variety of economic and security conditions at origin, allowing an investigation of variations in the pattern of selection of asylum seekers coming from different countries.

These different conditions are key to describing the origin-specific pattern of selection that prevails for asylum seekers in Germany. Individuals from Afghanistan, Iraq, and Syria are likely to be in danger at home, but asylum seekers from the Balkan region left countries considered to be safe.^4^ The level of threats that can be encountered in the origin country largely determines the high (low) rates of acceptance of asylum applications from conflict-affected (Balkan) countries.^5^ Accordingly, this could lead to differences in the expected duration of stay in Germany, such that asylum seekers from Afghanistan, Iraq, and Syria have a longer time horizon in the host country, compared with asylum seekers from Albania and Serbia, who are legally entitled to stay at destination only until their applications are rejected, something that almost invariably occurs.^6^

Albanians and Serbians have not needed a visa to enter the European Union since 2010 and 2009, respectively, and this facilitates a legal entry into the Schengen area. Germany was among the countries fearing a surge in asylum applications from Albania and Serbia after the visa requirement was lifted (Bertoli and Fernández-Huertas Moraga 2015), although this surge did not immediately materialize. Serbians and particularly Albanians started applying for asylum in Germany in large numbers in 2015, when the surge in applications from conflict-affected countries resulted in major delays in the processing of asylum claims.^7^ The processing time possibly increased the expected return from lodging an application for Albanians and Serbians, given that they were legally protected from the risk of deportation while their applications were processed, and could get access to welfare benefits.^8^

Different expected durations of stay in Germany influence the pattern of selection of asylum seekers with respect to education, through the returns to education at destination that increase with the time spent since migration (Dustmann and Glitz 2011).^9^ The longer time horizon of individuals from conflict-affected countries would imply a favorable selection on education.^10^ By contrast, Balkan asylum seekers are more likely to be negatively selected because of their greater probability of staying temporarily in Germany. This pattern of selection is consistent with the high (low) migration costs faced by asylum seekers originating from conflict-affected (Balkan) countries. Liquidity constraints on the decision of individuals from Afghanistan, Iraq, and Syria to migrate drive a positive selection with respect to education, whereas Albanians and Serbians encounter low migration costs to move to Germany. Moreover, the migration history of the five selected countries could also play a role in the selection of asylum seekers. Large migration networks from Serbia in Germany before the asylum surge might have facilitated the arrival of asylum seekers from these countries by decreasing the migration costs, resulting in a more negative pattern of selection on education.

I explore these predictions on the selection of asylum seekers in a country-by-country analysis of original data on asylum seekers in Germany. Comprehensive characteristics of asylum seekers are obtained from a survey conducted jointly by the Institute for Employment Research (IAB); the Research Centre on Migration, Integration, and Asylum of the Federal Office of Migration and Refugees (BAMF); and the Socio-Economic Panel (SOEP) at DIW Berlin. The IAB-BAMF-SOEP Refugee Sample allows me to exploit a large set of cases, which includes 4,328 asylum seekers. The data are matched with surveys conducted in the origin countries. Relevant information is combined into country-specific samples, and the empirical analysis uses a logistic model to examine the selection of asylum seekers with respect to education. Individuals claiming asylum in Germany from Iraq and Syria are shown to be positively selected on education, and the results provide mixed evidence on the selection of asylum seekers from Afghanistan. On the other hand, Albanian and Serbian asylum seekers are found to be drawn from the lower tail of the education distribution.

This article is related to various strands of the migration literature, in which the self-selection of immigrants has been widely studied, albeit rarely in the case of asylum seekers. Building on the idea that observable and unobservable characteristics influence the (pecuniary) benefits of migration, Borjas (1987, 1991) extended the Roy (1951) model to determine which individuals find migrating optimal. This seminal work was followed by several other contributions (Chiquiar and Hanson 2005; Chiswick 1999; Grogger and Hanson 2011). The implications derived from the Roy-Borjas model have been empirically studied for economic migrants in a variety of migration scenarios. Beginning with Chiquiar and Hanson (2005), analyses on the selection of immigrants from Mexico to the United States (Fernández-Huertas Moraga 2011; Kaestner and Malamud 2014; McKenzie and Rapoport 2010) and from different origin countries to OECD member states (Belot and Hatton 2012; Brücker and Defoort 2009; Mayda 2010) have flourished. More recently, Aksoy and Poutvaara (2019) extended the Roy-Borjas framework to account for the risks associated with conflicts or persecution. Their model shows that migrants from countries experiencing a major conflict are expected to be positively selected, even when the returns to skill at origin would be higher than in destination countries. Borrowing constraints strengthen the positive pattern of selection: individuals with more education are likely to have more resources and to be willing to leave in times of crises. To date, little is known about the pattern of selection of individuals who left their country of origin to seek asylum abroad. Birgier et al. (2016) provided evidence on the selection of political refugees fleeing Argentina and Chile during the military regimes there (1976–1983 and 1973–1985, respectively) to the United States, Sweden, and Israel. They documented that the decision process of these refugees regarding the choice of their destination is similar to those of economic immigrants. The descriptive work of Buber-Ennser et al. (2016) on individuals who arrived in Austria in 2015, mainly originating from Afghanistan, Iraq, and Syria, documented that the educational level of these asylum seekers was high relative to the average level of education in the origin countries. However, the analysis was hindered by representativeness issues regarding some of the data used and by the fact that individual-level information about asylum seekers in Austria was compared only with aggregate data of the origin population. More closely related to the current study, Lange and Pfeiffer (2018) evaluated the human capital selection of male asylum seekers in Germany. Their results suggested a positive selection of asylum seekers from Middle Eastern and African countries, who had 22% more years of schooling than the same-aged individuals in the origin country. The main difficulty of this study is the local dimension of the survey of asylum seekers, which implies that collected information is not representative of the asylum population in Germany. I contribute to the literature through the use of individual-level and nationally representative data for both the asylum seekers in Germany and the home-country population. Empirical support provided by Aksoy and Poutvaara (2019) highlights the favorable selection of refugees and complements my findings for other asylum destination countries. Their main contribution is to extend the analysis to other destination (or transit) countries of the recent refugee arrival in Europe. Focusing on Germany, I am able to depict differences in the selection on education *within* the refugee population in the host country, and I attempt to provide an interpretation of the observed patterns. Last, a recent work by Borjas and Monras (2017:376) noted that conditions at destination may influence the pattern of selection of asylum seekers. In line with this argument, I argue that the origin-specific expected length of stay in Germany is likely to explain differences in the selection of asylum seekers with respect to education.

In addition to contributing to the self-selection literature, this analysis extends work on the determinants of asylum applications to developed countries (Hatton 2009, 2016; Neumayer 2004, 2005; Thielemann 2006;). Instead of using aggregate information that allows a focus on only the sheer scale of asylum applications, my study relies on survey data to identify the characteristics of individuals seeking asylum in Germany. As a result, I am able to evaluate who migrates from the main asylum source countries rather than analyzing the macroeconomic forces that trigger the migration of asylum seekers.

## Selected Countries of Origin and Data Sources

### Selected Countries of Origin

**Fig. 1:**
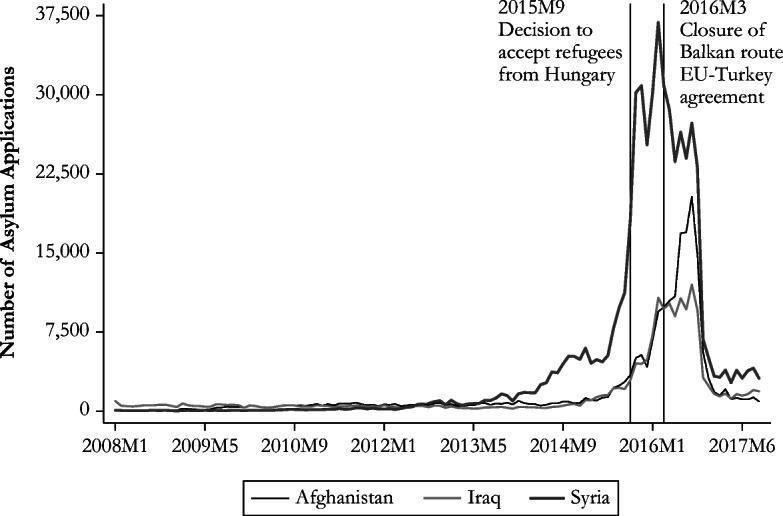
Asylum applications in Germany from conflict-affected countries. *Source*: Author’s elaboration based on Eurostat (2017b).

**Fig. 2:**
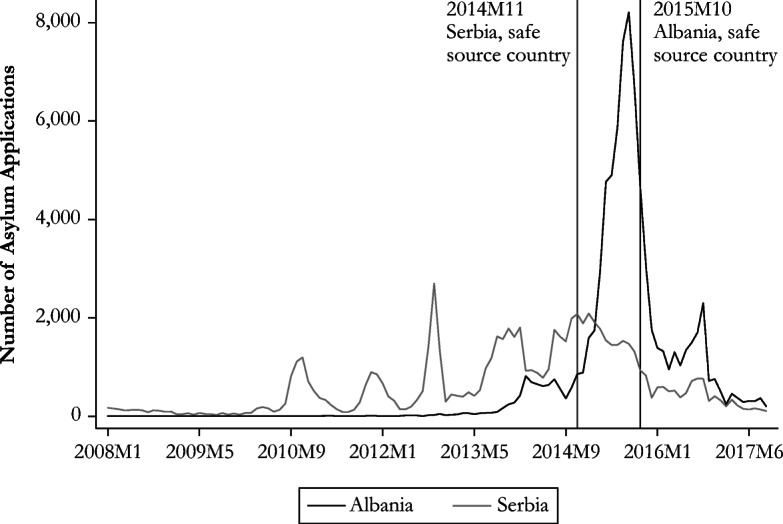
Asylum applications in Germany from Balkan countries. *Source*: Author’s elaboration based on Eurostat (2017b).

The recent evolution of the number of asylum applications lodged in Germany has implied several changes in the German asylum policy and raised interest in studying the characteristics of the newcomers. The civil conflict in Syria forced the migration of 5.5 million Syrians, and most asylum seekers who were able to move to Europe went to Germany (United Nations High Commissioner for Refugees (UNHCR) 2016). The related surge in the number of Syrian asylum applications is reflected in Fig. 1. Germany also experienced a large influx of asylum seekers fleeing turmoil in Afghanistan and Iraq (also shown in Fig. 1) as well as asylum seekers from the Balkan region (Fig. 2). However, the pattern is remarkably different between Albania and Serbia. The evolution of asylum applications from Albania is similar to the one of conflict-affected countries, whereas asylum claims from Serbia were more evenly spread (between 0 and 2,000 applications) over a longer period.

This large influx of asylum seekers has prompted several changes in the asylum policy of destination countries. European countries closed the Western Balkan route (March 9, 2016) and implemented an agreement with Turkey shortly thereafter (March 18, 2016). The latter aimed to address the overwhelming arrival of smuggled asylum seekers going across the Aegean Sea from Turkey to the Greek islands, by allowing Greece to deport to Turkey “all new irregular migrants” (European Council 2016) arriving since March 20, 2016. In return, EU member states agreed to increase the resettlement of Syrian refugees residing in Turkey, enhance visa liberalization for Turkish nationals, and expand existing financial support for the refugee population in Turkey. These decisions can certainly explain the downward slope in the number of applications beginning in mid-2016. At the national level, German authorities reacted to the inflow of asylum seekers from the Balkan region by repeatedly modifying its list of safe countries of origin.^11^ Serbia was included in November 2014 (along with the Republic of Macedonia and Bosnia-Herzegovina),^12^ and Albania was added in October 2015 (with Kosovo and Montenegro).^13^ This policy change triggered a decrease in the number of asylum claims from these two countries.

Asylum seekers in Germany are mainly from conflict-affected countries (i.e., Afghanistan, Iraq, Syria) and the Balkan region (Albania, Serbia, Kosovo), but also come from a few other countries (e.g., Eritrea, Somalia, Iran, and Pakistan). The sheer scale of the asylum surge is shown in Table 1, which reports the number of asylum seekers across origins recorded by the Federal Office for Migration and Refugees (BAMF) between the beginning of 2013 and the end of January 2016. As a consequence of the ongoing crisis in Syria, 41.5% of the asylum seekers originate from this country; individuals from Afghanistan and Iraq correspond, respectively, to 9.8% and 8.3% of asylum seekers.

**Table 1:** Composition of the recent arrival of asylum seekers in Germany

	Asylum Seekers (AZR)	Asylum Seekers (IAB-BAMF-SOEP)
Total	529,078	4,328
	(100.0)	(100.0)
Syria	219,673	2,181
	(41.5)	(42.6)
Afghanistan	51,709	527
	(9.8)	(13.6)
Iraq	44,138	538
	(8.3)	(8.7)
Albania, Serbia	31,104	164
	(5.9)	(3.8)
Others	182,454	918
	(34.5)	(31.3)

*Notes:* The first column represents cases in the register of foreigners (AZR) at BAMF, for whom the entry in Germany occurred between January 1, 2013, and January 31, 2016. The second column corresponds to asylum seekers surveyed in the IAB-BAMF-SOEP Refugee Sample. Numbers for Albania and Serbia also include Kosovo. Shares by column are reported in parentheses, and for the IAB-BAMF-SOEP Refugee Sample are weighted to be representative.

*Sources:* Author’s calculations based on Brücker et al. (2016) and IAB-BAMF-SOEP Refugee Sample.

The self-selection of asylum seekers from the origin population is examined for a limited number of source countries: Afghanistan, Albania, Iraq, Serbia, and Syria. These five countries represent roughly 65% of all recent asylum seekers in Germany (Table 1). Moreover, they offer an interesting variety with respect to economic and security conditions at origin, which lead to differences in the migration costs and the origin-specific duration of stay in Germany. These differences, in turn, are likely to affect the observed pattern of selection of asylum seekers.

### Individual-Level Data

#### *A Survey of Asylum Seekers in Germany*

The IAB-BAMF-SOEP Refugee Sample is used to extract comprehensive information for individuals who fled their home country to seek asylum in Germany. The study surveyed recently arrived asylum seekers on a broad range of topics and included questions on their socioeconomic attributes, migration experience, past and current living conditions, and labor market experience as well as attitudes about some sociopolitical issues (democracy, religion, and gender equality). I rely on the first wave of the survey, which was conducted in 2016 and covers 4,328 adult asylum seekers who arrived in Germany since 2013.

The sample was drawn from the Central Register of Foreigners (AZR) of the BAMF, making the survey representative of asylum seekers who arrived in Germany between January 1, 2013, and January 31, 2016, and were registered as asylum seekers by the end of June 2016 (for details on the design, methodology, and response rate of the survey, see Kroh et al. 2017). Individuals with a higher likelihood of being granted refugee status in Germany at the time of the sampling (i.e., those from Afghanistan, Iraq, and Syria), women, and persons over age 30 were oversampled. Given this oversampling, I use appropriate weighting methods so that the results can be interpreted as representative of the asylum population.

#### *Country-Specific Surveys of the Origin Population*

This section proposes a brief overview of the data combined with information about asylum seekers in Germany to build the origin-specific samples required to carry out the empirical analysis (for descriptive statistics for each survey of the origin population, see online appendix section A1). Two important comments should be made regarding the surveys conducted in the asylum source countries under focus. On the one hand, because the situation in Syria makes it difficult (if not impossible in some areas) to conduct surveys, I must rely on data collected in 2006, before the surge of asylum seekers in Germany.^14^ On the other hand, the five samples are representative of the national origin population, and this holds regardless of the main purpose of each survey.^15^ The representativeness is key and allows me to assess the selection of asylum seekers by avoiding potential biases that could arise if one were to compare the recent asylum seekers in Germany with a selected group at origin.

#### *Afghanistan, Iraq, and Syria*

Information about the origin population for Afghanistan comes from the Asia Foundation, which conducted the Survey of the Afghan People (SAP) yearly from 2004 to 2016. The SAP is a public opinion survey that explores social, economic, and political issues in Afghanistan. The study has gathered the opinions of more than 87,000 persons, providing an interesting portrait of individual perceptions and their evolution over time. I pool six recent waves (2011–2016) to build the sample of individuals who have stayed in Afghanistan.

Data for Iraq are drawn from the Living Standards Measurement Study (LSMS) of the World Bank (Institute of Statistics of Albania 2012). More specifically, I exploit the Household Socio-Economic Survey (HSES), which was implemented for the second time in Iraq in 2012–2013 (Organization for Statistics and Information Technology (COSIT) and Kurdistan Regional Statistics Office (KRSO) 2012–2013). The main objective of the study is to provide information to measure and analyze poverty throughout the country, but it also evaluates the socioeconomic situation of individuals in Iraq. The total sample size is 24,944 households, which corresponds to 176,042 individuals.

Individual-level data on the Syrian population are rarely available, particularly for recent years. I am nonetheless able to derive representative information from UNICEF’s 2006 Multiple Indicator Cluster Survey (MICS; Central Bureau of Statistics 2006). The primary goal of the survey is to deliver insights on the situation of children and women in Syria, but I can extract some relevant socioeconomic characteristics for this study. UNICEF successfully interviewed 19,870 households, among which 107,365 individuals were listed. Of the full sample, I keep only 55,277 observations because of restrictions on the age of individuals (18–64); the survey involved a large number of individuals younger than age 18. The data cover 28,297 men and 26,980 women, among whom 49% and 46.1%, respectively, are aged 18–30.

#### *Albania and Serbia*

The LSMS of the World Bank is also the data source for Albania. This multipurpose study, which aimed to measure and evaluate the living conditions and the poverty situation in the country, was conducted several times (2002, 2003, 2004, 2005, 2008, and 2012). I use data from the last round of the survey (i.e., 2012), in which 6,671 households and a total of 25,335 individuals were interviewed. The sample contains 16,108 cases, with 8,084 men and 8,024 women, and respective shares of individuals aged 18–30 of 35.1% and 30.2%.

Finally, information for the origin population of Serbia is obtained from the European Union Statistics on Income and Living Conditions (EU-SILC). Surveys in Serbia have been administered since 2013, and the 2013–2015 waves are pooled to form the sample under focus (EU-SILC 2013–2015). The EU-SILC provides data on income, poverty, social exclusion, and living conditions, and it is specifically designed to be suitable for comparative statistics across European countries. At the individual level, data on the socioeconomic attributes and the labor market characteristics of the interviewees are available.

### Harmonization of Data Sources

The aforementioned data are combined to build five origin-specific samples. These samples are the result of the matching of information of the population in the source countries and the asylum seekers surveyed in Germany. The related harmonization is straightforward for several sociodemographic factors (e.g., age, gender, and marital status) given that they are commonly defined and measured across the different surveys. However, this procedure is more demanding and time-consuming for the level of education and the perceived level of insecurity in the home country. Section A2 of the online appendix provides details on the methodology followed to link available information between the various data sources. The final number of observations in each sample is given in Table 2.^16^ For instance, the final sample for Syria is composed of 54,014 individuals, among whom 3.8% are asylum seekers who recently arrived in Germany.

**Table 2:** Size of the respective origin-specific samples by migration status

	Afghanistan	Iraq	Syria	Albania	Serbia
Origin Population	50,406	80,722	51,968	14,829	33,395
	(99.1)	(99.4)	(96.2)	(99.7)	(99.8)
Asylum Seekers	442	485	2,046	46	43
	(0.9)	(0.6)	(3.8)	(0.3)	(0.1)
Total	50,848	81,207	54,014	14,875	33,438
	(100.0)	(100.0)	(100.0)	(100.0)	(100.0)

*Note:* Respective shares are reported in parentheses.

*Sources:* Author’s calculations based on SAP (2011–2016), COSIT and KRSO (2012–2013), Central Bureau of Statistics (2006), Institute of Statistics of Albania (2012), EU-SILC (2013–2015), and IAB-BAMF-SOEP Refugee Sample.

## Empirical Analysis

The combination of individual-level data for the five countries under focus paves the way for an empirical analysis of the characteristics that shape the selection of asylum seekers from the origin population. The set of variables considered in each country-specific sample is described in section A3 of the online appendix, and weighted summary statistics are presented in section A4. The study aims to shed light on the self-selection of asylum seekers with respect to education. Therefore, I mainly present and discuss findings related to differences in the observed level of education between asylum seekers and the home country population.

### Descriptive Evidence

The country-specific distributions of education of the population at origin and the asylum seekers in Germany are shown in Table 3. The pattern of selection of asylum seekers from conflict-affected countries seems to be positive: the share who attended tertiary education is higher among asylum seekers than among their counterparts in the origin population. In the case of Syria, the figures reveal that 16.5% of asylum seekers are highly educated, compared with only 5.7% of the home country population. On the other hand, asylum seekers from Albania and Serbia appear to be negatively selected with respect to education. In the case of Serbia, only 4.9% of individuals in the origin population did not attend more than primary education, but the share peaks at 81.4% for asylum seekers.^17^

**Table 3:** Origin-specific distribution of education by migration status

	Afghanistan		Iraq		Syria		Albania		Serbia	
	Origin	Asylum	Origin	Asylum	Origin	Asylum	Origin	Asylum	Origin	Asylum
Primary or Less	77.6	74.7	77.1	71.1	52.5	52.5	48.6	58.7	4.9	81.4
Secondary	20.0	19.0	18.0	17.9	41.8	31.0	36.7	39.1	77.7	18.6
Tertiary	2.4	6.3	4.9	10.9	5.7	16.5	14.8	2.2	17.4	
Total	100.0	100.0	100.0	100.0	100.0	100.0	100.0	100.0	100.0	100.0
Chi-Square Test		27.5**		37.7**		434.4**		6.0*		533.5**
Likelihood Ratio Test		19.4**		28.6**		322.4**		8.8*		173.6**

*Notes:* Reported figures correspond to the share of individuals in each cell. Weighted statistics can be found in the summary statistics presented in section A4 of the online appendix. Chi-Square Test is the test of independence, and Likelihood Ratio Test is the likelihood-ratio test for proportions.

*Sources:* Author’s calculations based on SAP (2011–2016), COSIT and KRSO (2012–2013), Central Bureau of Statistics (2006), Institute of Statistics of Albania (2012), EU-SILC (2013–2015), and IAB-BAMF-SOEP Refugee Sample.

*p < .05; **p < .01

The last two rows of Table 3 present the statistics related to the test of independence (chi-square test) and the likelihood-ratio test for proportions. These tests are used to compare the country-specific distribution of education of the origin population with that of asylum seekers. The results indicate that the two distributions are significantly different with respect to education for all origins considered in the analysis.

The IAB-BAMF-SOEP survey includes questions about the self-assessed relative income and economic position of asylum seekers relative to the home country population. The related statistics are introduced in Table 4 for each country of origin and show that 19% to 29% of asylum seekers originating from the three conflict-affected countries self-report being better-off (i.e., above average in both dimensions) compared with the origin population. This provides evidence of a positive pattern of selection with respect to their economic situation before their migration to Germany. By contrast, asylum seekers from Balkan countries come from the lower end of the income distribution, as suggested by the fact that 76% to 87% of Albanians and Serbians seeking asylum in Germany self-assess their economic position as being below the average of the home country population.

**Table 4:** Self-assessed income and economic position relative to the home country population

	Afghanistan		Iraq		Syria		Albania		Serbia	
	Income	Economic Position	Income	Economic Position	Income	Economic Position	Income	Economic Position	Income	Economic Position
Below Average	29.5	21.6	29.9	25.3	34.2	17.9	76.0	76.9	87.3	85.3
Average	48.8	50.1	50.9	56.0	43.3	53.0	21.0	20.7	12.7	14.7
Above Average	21.7	28.4	19.2	18.7	22.5	29.1	3.0	2.4	0.0	0.0
Total	100.0	100.0	100.0	100.0	100.0	100.0	100.0	100.0	100.0	100.0

*Notes:* Reported figures correspond to the weighted share of asylum seekers in each cell. Income refers to the following question: “If you compare your net income at that time with the income of other people in your country, how would you describe your level of net income there?” Economic Position pertains to the following question: “How would you estimate your financial situation at that time with the income of other people in your country?” For each question, five answers were available: (1) well above average, (2) above average, (3) average, (4) below average, and (5) well below average. I group (1) and (2) in the “above average” category, while (4) and (5) are grouped in the “below average” category.

*Source:* Author’s calculations based on IAB-BAMF-SOEP Refugee Sample.

The pattern of selection observed for the three conflict-affected countries refers to asylum seekers who were able to flee their home country and successfully reached Germany. However, only a tiny fraction of all asylum seekers managed to arrive in Europe. More than 300,000 asylum seekers in Germany come from Afghanistan, Iraq, or Syria (Table 1). At the European scale, this figure is high, but it is not high compared with asylum seekers hosted by neighbors of the main asylum source countries (Fig. 4; UNHCR 2016:15). The actual difference in the number of asylum seekers suggests that the recent asylum population in Germany is likely to represent a selected subsample of all asylum seekers who were able to leave their origin country. More specifically, it raises questions about whether the pattern of selection depends on (1) the selection of asylum seekers who left their home country or (2) the selection of asylum seekers who managed to go to Germany among those who fled Afghanistan, Iraq, or Syria. In other words, can the pattern of selection be extended for conflict-affected countries to other asylum seekers who ran away from their origin country without migrating to Germany? Based on information collected in the fourth wave of the Arab Barometer (2018), we can evaluate the distribution of education of Syrian refugees who have migrated to Jordan and Lebanon. In Jordan, the share of refugees with primary education or less is 46%, but the share of tertiary-educated refugees is 8.3%. In Lebanon, the shares of low-educated and high-educated refugees are 57.3% and 6.3%, respectively. These figures suggest that refugees in Jordan are slightly positively selected (to a lower extent than Syrian asylum seekers in Germany), whereas refugees in Lebanon are relatively similar to the education profile of the origin population (Table 3). Notice, however, that other data sources (e.g., Verme et al. 2016) have revealed a different pattern of selection on education for Syrian refugees who fled to Lebanon and Jordan. All things considered, the results outlined in this article are likely not to apply to the entire population of forced migrants, indicating that asylum seekers in Germany may represent a selected subsample of this population.

### Empirical Strategy and Results

Collected information can be used to study the self-selection of asylum seekers with respect to education while other characteristics that can affect the pattern of selection are controlled for. The empirical strategy relies on the estimation of origin-specific logistic regressions with the following specification:

1 $$ P\left({Y}_{ij}=1\left|{\mathbf{X}}_{ij}\right.\right)=\frac{\exp \left({\upbeta}_j^{\prime }{\boldsymbol{X}}_{ij}\right)}{1+\exp \left({\upbeta}_j^{\prime }{\boldsymbol{X}}_{ij}\right)}, $$

where *Y*_*ij*_ is a binary indicator taking the value 1 if an individual *i* left her home country *j* to seek asylum in Germany, and 0 otherwise. **X**_*ij*_ represents individual attributes of asylum seekers: (pre-migration) level of education; age; age squared; gender; marital status; and, sporadically, perceptions about security conditions at origin, (pre-migration) ability to speak German, information about religion, and occupational status before migration. Notice that the set of covariates changes for each country-specific estimation because of differences in the availability of data across the surveys of the origin population.^18^

The results are presented through both the predicted probabilities of seeking asylum in Germany for each level of education and the average marginal effects, which are calculated for each individual with their observed values of covariates and then averaged across all individuals. The estimates are displayed for the three conflict-affected countries (Table 5) and for the two Balkan countries (Table 6); section A6 in the online appendix reports the standard coefficients. The level of education of asylum seekers in Germany is evaluated with respect to the distribution of education of the origin population. In each sample, the variable is divided into three levels of education: primary or less, secondary, and tertiary education. High-educated individuals (i.e., individuals who attended tertiary education in the home country) represents the benchmark category for all countries except Serbia, which has individuals with secondary or more education as reference group, and the average marginal effects are interpreted accordingly.

**Table 5:** Self-selection of asylum seekers from conflict-affected countries^a^

	Afghanistan			Iraq		Syria		
	(1)	(2)	(3)	(4)	(5)	(6)	(7)	(8)
Probability of Migrating								
Level of education								
Primary or less	.009**	.009**	.009**	.006**	.006**	.041**	.041**	.041**
	(.001)	(.001)	(.001)	(.000)	(.000)	(.001)	(.001)	(.001)
Secondary	.006**	.007**	.007**	.005**	.006**	.026**	.026**	.026**
	(.001)	(.001)	(.001)	(.001)	(.001)	(.001)	(.001)	(.001)
Tertiary	.017**	.018**	.014**	.011**	.014**	.090***	.087**	.087**
	(.003)	(.004)	(.003)	(.002)	(.002)	(.005)	(.005)	(.005)
Average Marginal Effects								
Level of education								
Primary or less	–0.008*	–0.009*	–0.005	–0.006**	–0.008**	–0.049**	–0.046**	–0.045**
	(0.003)	(0.004)	(0.003)	(0.002)	(0.002)	(0.005)	(0.005)	(0.005)
Secondary	–0.011**	–0.012**	–0.007*	–0.006**	–0.008**	–0.064**	–0.061**	–0.060**
	(0.003)	(0.004)	(0.003)	(0.002)	(0.002)	(0.005)	(0.005)	(0.005)
Age	0.000**	0.000**	0.000**	0.000	–0.000^†^	0.000*	0.000	0.000
	(0.000)	(0.000)	(0.000)	(0.000)	(0.000)	(0.000)	(0.000)	(0.000)
Male	0.004**	0.005**	0.004**	0.003**	0.003**	0.020**	0.019**	0.019**
	(0.001)	(0.001)	(0.001)	(0.001)	(0.001)	(0.002)	(0.002)	(0.002)
Married	–0.010**	–0.009**	–0.009**	–0.003**	–0.003**			
	(0.001)	(0.001)	(0.001)	(0.001)	(0.001)			
Insecurity		0.018**	0.018**		0.022**			
		(0.002)	(0.002)		(0.002)			
Speaks German			0.038**					
			(0.004)					
PTS^b^							0.094**	
							(0.005)	
FH CL^c^								0.155**
								(0.008)
Number of Observations	50,848	50,848	50,848	81,207	81,207	54,014	54,014	54,014
McFadden’s *R*^2^	.028	.074	.097	.018	.103	.038	.146	.164

*Notes:* All models are estimated using logistic regressions. McFadden’s *R*^2^ = 1 − ln(*L*_*M*_) / ln(*L*_0_), where *L*_*M*_ is the likelihood of the estimated model, and *L*_0_ is the likelihood of the model without predictors. Robust standard errors are shown in parentheses.

*Sources:* Author’s calculations based on SAP (2011–2016), COSIT and KRSO (2012–2013), Central Bureau of Statistics (2006), Political Terror Scale from Gibney et al. (2017), Freedom House (2017), and IAB-BAMP-SOEP Refugee Sample.

^a^Dependent variable = 1 if an individual has migrated, 0 otherwise. Benchmark group = tertiary education.

^b^PTS corresponds to Political Terror Scale.

^c^FH CL is the Civil Liberties index from the Freedom House.

^†^*p* < .10; *p < .05; **p < .01

**Table 6:** Self-selection of asylum seekers from Balkan countries^a^

	Albania			Serbia		
	(1)	(2)	(3)	(4)	(5)	(6)
Probability of Migrating						
Level of education						
Primary or less	.004**	.004**	.004**	.045**	.020**	.126**
	(.001)	(.001)	(.001)	(.008)	(.003)	(.018)
Secondary	.003**	.003**	.003**	.000**	.000**	.001**
	(.001)	(.001)	(.001)	(.000)	(.000)	(.000)
Tertiary	.000	.000	.000			
	(.000)	(.000)	(.000)			
Average Marginal Effects						
Level of education						
Primary or less	0.003**	0.004**	0.004**	0.045**	0.020**	0.125**
	(0.001)	(0.001)	(0.001)	(0.008)	(0.003)	(0.019)
Secondary	0.003**	0.003**	0.003**			
	(0.001)	(0.001)	(0.001)			
Age	–0.000*	–0.000**	–0.000*	–0.000**	–0.000**	–0.000**
	(0.000)	(0.000)	(0.000)	(0.000)	(0.000)	(0.000)
Male	–0.001	–0.001	–0.001	0.001^†^	0.001^†^	0.004*
	(0.001)	(0.001)	(0.001)	(0.000)	(0.000)	(0.001)
Married	0.002	0.002	0.002	0.002**	0.001**	0.007**
	(0.002)	(0.002)	(0.002)	(0.000)	(0.000)	(0.002)
Speaks German		0.010**				
		(0.002)				
Religion						
Atheist			0.026^†^			
			(0.014)			
Orthodox/other			0.001			
			(0.002)			
Catholic			0.001			
			(0.002)			
Occupation						
No work					0.001**	
					(0.000)	
Worker					0.008**	
					(0.001)	
Self-employed					0.001	
					(0.001)	
Insecurity						–0.001
						(0.001)
Number of Observations	14,875	14,875	14,875	33,438	33,438	8,400
McFadden’s *R*^2^	.054	.074	.073	.435	.515	.518

*Notes:* All models are estimated using logistic regressions. McFadden’s *R*^2^ = 1 *−* ln(*L*_*M*_) / ln(*L*_0_), where *L*_*M*_ is the likelihood of the estimated model, and *L*_0_ is the likelihood of the model without predictors. Robust standard errors are shown in parentheses. Muslim is the benchmark category to analyze the religious affiliation of Albanians. Employee (both with and without supervision tasks) is the reference group to interpret the occupational status in Serbia. Information about insecurity in Serbia is available only in the 2013 wave, which explains the number of observations reported in column 6.

*Sources:* Author’s calculations based on Institute of Statistics of Albania (2012), EU-SILC (2013–2015), and IAB-BAMF-SOEP Refugee Sample.

^a^Dependent variable = 1 if an individual has migrated, 0 otherwise. Benchmark group = tertiary education for Albania, and secondary education or more for Serbia.

^†^*p* < .10; *p < .05; **p < .01

The first three columns of Table 5 provide evidence of a positive selection on education for asylum seekers from Afghanistan. The average marginal effects are negative and significant, indicating that asylum seekers in Germany are more likely to be highly educated than those who stayed in Afghanistan. This pattern of selection is consistent with the assumption that asylum seekers originate from a better-off subsample of the Afghan population. This, in turn, could reflect the fact that only certain individuals can afford the relatively high migration costs required to migrate to Germany. By contrast, poorer Afghans might have ended up in neighboring countries or remained at home. Moreover, the favorable selection of asylum seekers persists when the subjective perceptions about the level of insecurity (column 2) and the retrospective language proficiency in German (column 3) are added into the specification. The positive coefficient of the former suggests that asylum seekers left the country because they felt more unsafe at home than their nonasylum counterparts. This result is in line with the literature on the (macroeconomic) determinants of asylum migration, which shows that higher values of the Political Terror Scale and less individual freedom (Freedom House) push individuals out of their origin country (Hatton 2009, 2016).

The selection on education of asylum seekers from Iraq is detailed in columns 4 and 5 of Table 5. The results reveal a positive pattern of selection of asylum seekers with respect to the origin population. More specifically, the estimates in column 5 imply that the probability of seeking asylum in Germany is 1.4% for individuals with tertiary education but only 0.6% for individuals with a primary education or less. Consequently, the average marginal effects are negative and significant, showing that Iraqi asylum seekers have a lower likelihood of being low- and secondary-educated relative to those who remained in Iraq. Similar to Afghanistan, the observed selection could be explained by the high migration costs needed to reach Germany, and asylum seekers have been forced to flee Iraq because they feared for their own security.

The analysis focuses then on the pattern of selection of asylum seekers from Syria, and the last three columns of Table 5 document the relevant probabilities and average marginal effects. They all depict a positive selection of asylum seekers with respect to premigration education. The probability of migrating is 8.7% for high-educated individuals—more than twice the likelihood of seeking asylum in Germany for low-educated individuals (4.1%). Differences across education groups can be shown with the average marginal effects. They highlight that the probability of claiming asylum in Germany decreases by 4.5 to 6.4 percentage points for individuals with low or secondary education, compared with high-educated individuals. Consistent with the literature, asylum seekers have been pushed out of Syria by a greater level of political terror and a worsening of civil liberties in their home country.^19^

The results show a favorable pattern of selection on education for asylum seekers originating from conflict-affected countries. By contrast, the findings are strikingly different for asylum seekers from the Balkan region. Both Albanians and Serbians who recently arrived in Germany via the asylum channel are negatively selected on education, as suggested by the estimates reported in Table 6. On the one hand, the probability of migrating to Germany for low- and secondary-educated individuals is positive and significant, but that for high-educated individuals from Albania is insignificant. On the other hand, the average marginal effects indicate that the differences in the probability of migrating are positive and significant for asylum seekers from Albania with low and secondary education and for low-educated asylum seekers from Serbia. Besides information on education, I am also able to take into account other characteristics in the specifications. The negative pattern of selection of Albanian asylum seekers still prevails when the retrospective ability to speak German (column 2) or religious affiliation (column 3) are included in the list of covariates. Serbian individuals seeking asylum in Germany tend to have held (in the origin country) positions as a worker rather an employee, compared with individuals who have no work experience (column 5). Controlling for this variable, however, mitigates the observed negative selection of asylum seekers from Serbia. Last, the decision to migrate taken by Serbian asylum seekers is not influenced by the perceived level of insecurity in the home country (column 6). This finding supports the idea that they did not leave Serbia because they were threatened there and could potentially reinforce the fact that Serbia can be considered as a safe source country. This outcome clearly contrasts with the conclusions for asylum seekers from Afghanistan, Iraq, and Syria.

### Robustness Checks

This section presents the results obtained from the estimation of the baseline specifications with an alternative estimator and different country-specific subsamples. These results confirm the conclusions derived for all countries except Afghanistan, for which estimates are found to be sensitive to sample selection.

#### *Selection of Asylum Seekers Through the Analysis of Rare Events*

The study is based on country-specific information, and the relative number of asylum seekers in some of the origin-specific samples (i.e., Albania and Serbia) is small (Table 2). Thus, the maximum likelihood estimation of the logistic model might suffer from small-sample biases. To ensure that the results are not affected by this issue, I estimate the fit models with penalized maximum likelihood estimation following the methodology proposed by Firth (1993). This procedure leads to the average marginal effects compiled in Table A7.1 in the online appendix. These effects are highly similar to the estimates obtained in the Empirical Strategy and Results section, ruling out potential biases affiliated with the low number of individuals seeking asylum in Germany contained in the dependent variable.

#### *Selection With Respect to Urban/Rural Origin Population*

The benchmark analysis does not control for potential information about the place of departure of asylum seekers in the origin country. However, the positive pattern of selection of asylum seekers from conflict-affected countries might be driven by the fact that they fled urban areas, which could on average host more high-educated individuals. The reverse occurs for Balkan countries, and the negative selection might be the consequence of asylum seekers originating from rural areas, where the average level of education is likely to be lower than in cities.

This question cannot be directly evaluated because the IAB-BAMF-SOEP Refugee Sample does not include data on the starting point of migration from the home country to Germany. On the other hand, the origin-specific surveys allow me to determine whether individuals are located in an urban or a rural area. One way to address the lack of information relative to asylum seekers is to assume that all those who are from conflict-affected (Balkan) countries come from urban (rural) locations in their source country. The empirical study is then replicated to check whether the observed pattern of selection is the result of the selection of asylum seekers with respect to the urban/rural composition of the origin population. The related average marginal effects are presented in Table A7.2 in the online appendix.

The positive (negative) pattern of selection documented for asylum seekers from Iraq and Syria (Albania and Serbia) is not altered when their level of education is compared with that of individuals who live in urban areas. By contrast, Afghan asylum seekers are now negatively selected with respect to the urban origin population, and this raises questions on the robustness of the results reported in Table 5. However, the conservative assumption that all asylum seekers from Afghanistan who fled toward Germany are from urban locations might not be consistent with the urban/rural composition that prevails in the country. With only 25% (United Nations 2018) of the Afghan population living in urban areas in 2015, it is likely that some asylum seekers originate from rural areas and would better be compared with the rural population to correctly analyze the robustness of the pattern of selection on education.

#### *Self-selection of Male Asylum Seekers*

In the migration literature, the self-selection of migrants has been mainly studied among men or by dividing the sample into men and women. The origin-specific samples used in the baseline analysis did not make this distinction. The arrival of female asylum seekers in Germany might follow the initial departure of men, and this mechanism could distort the results obtained previously. Therefore, I reestimate the various specifications presented earlier by considering only male asylum seekers. The resulting average marginal effects, displayed in Table A7.3 in the online appendix, support the findings depicted with the full-sample estimates for all countries except Afghanistan, for which the education profile of asylum seekers with respect to the origin population appears to be sensitive to choices made about sample selection.

#### *Self-selection of Family-Tied Asylum Seekers*

All asylum seekers surveyed in the IAB-BAMF-SOEP Refugee Sample have been considered in the benchmark estimations. However, the probability of seeking asylum in Germany for some individuals might depend on their family ties, so the chosen destination could also be the residence of at least one of their relatives. Although no explicit question on family reunification has been asked of asylum seekers, those who are susceptible to migrating to Germany through the family channel are identified as having at least one of the following two characteristics: (1) they left the origin country because some of the family members moved abroad, and/or (2) they chose Germany because they have relatives already living there. Then, the analysis is reproduced with samples that involve only those individuals with potential family links in Germany to check for potential differences in the pattern of selection on education of family-tied asylum seekers.

The estimates for education are provided in Table A7.4 in the online appendix. All specifications have been replicated, but the baseline group has been switched from tertiary education to secondary education or more for Balkan countries because of constraints on the number of asylum seekers in the dependent variable. The results obtained with the origin-specific subsamples reveal that a positive selection on education still arises for asylum seekers from Iraq and Syria, whereas Serbian asylum seekers are negatively selected on education with respect to the origin population. Moreover, family-tied asylum seekers from Albania and Afghanistan are comparable in terms of education to their nonasylum counterparts.

## Interpretation of the Empirical Results

This section provides arguments and supportive evidence to understand the findings obtained in the econometric analysis. The main goal is to interpret the observed difference in the pattern of selection of asylum seekers from the two groups of origins.

Economic and security conditions differ across origin countries. Individuals from Afghanistan, Iraq, or Syria are likely to be threatened or persecuted at home. The individuals in Albania and Serbia, however, are not considered to be endangered, which explains why these two countries were included in the list of safe source countries. This difference has consequences on the probability of being granted the refugee status in Germany. Indeed, the recognition rate is relatively high for asylum seekers from conflict-affected countries but is extremely low for asylum seekers from the Balkan region. In 2015, 72.8% of asylum applications from Afghanistan were accepted by Germany, and the acceptance rates for Iraq and Syria were 98.3% and 97.7%, respectively. However, Germany approved only 0.2% and 0.1% of asylum claims from, respectively, Albania and Serbia (Eurostat 2018).

Origin countries also differ in terms of migration costs. More specifically, the median total cost of migration (i.e., the sum of the costs associated with transport, accommodation, and smuggling) is 2,015 euros for conflict-affected countries but is only 280 euros for Balkan countries. Moreover, the median time to reach Germany from conflict-affected countries is 23 days, compared with only 2 days for Balkan countries.^20^ The lower figures for asylum seekers from Albania and Serbia highlight that the door was rather open between the Balkan region and Germany. Higher metrics for asylum seekers from Afghanistan, Iraq, and Syria imply that it was more difficult for them to reach the host country.

Both the migration costs and the origin-specific asylum recognition rate are consistent with differences in the pattern of selection of asylum seekers who recently arrived in Germany. The higher costs and acceptance rates faced by asylum seekers from conflict-affected countries would lead to a more positive selection on education. On the one hand, savings are likely to be positively correlated with skills, such that the liquidity constraints on the decision to migrate would determine a positive pattern of selection. On the other hand, asylum seekers from unsafe areas have a higher recognition rate, which allows them to expect to stay longer (or even permanently) in Germany. Because transferring human capital across borders takes time, the returns to education at destination is an increasing function of the time spent there (Dustmann and Glitz 2011). Even though asylum seekers might enjoy limited returns to education on the German labor market in the early stages of their stay, the time horizon could be sufficiently long for the income gains from migration to become an increasing function of education.

Asylum seekers from Balkan countries encounter lower costs of migration and are able to enter the destination without a visa, enhancing the attractiveness of migrating to Germany. However, Germany considers Albania and Serbia to be safe. This implies that the probability of acceptance of asylum claims is close to zero and that, after an asylum claim is denied, asylum seekers can either leave the host country (voluntarily or by force) or stay in Germany as undocumented migrants.^21^ In principle, this should compel them to remain only temporarily in Germany; in fact, the limited legal time refers to the period required to process the asylum applications. Misusing the asylum channel as a legal temporary migration scheme might exclusively be attractive for low-educated individuals, such that the income gains from migration are a decreasing function of education. If the asylum seekers decide to remain as undocumented migrants, they could stay longer in Germany but would be able to work only in the informal sector, where the returns to education are lower than in the formal labor market.^22^ This would also coincide with a negative pattern of selection of asylum seekers with respect to education.

The perspective from being able to stay only temporarily in the receiving country raises questions about whether the time taken to process the applications could be beneficial for asylum seekers from Albania or Serbia. First, they are protected from deportation to their home country during the claim processing time. Because of the insufficient capacity of German authorities to process the surge of asylum applications in 2015, the number of pending cases increased sharply, mechanically increasing the time needed to process these claims.^23^ However, priorities given to process the claims from some origin countries might have resulted in differences in the expected processing time across countries.

To explore this idea, I compute the origin-specific expected processing time of asylum applications in Germany.^24^ The average time to determine whether the request would be accepted in 2015 was high for Afghanistan (25 months) and Iraq (15.5 months), whereas Syrian asylum claims were processed more quickly (4.5 months). The figures for Albania (9 months) and Serbia (15.5 months) indicate that the expected time to process the asylum claims was high when Balkan asylum seekers arrived in Germany. Recall that this metric corresponds to the temporary legal period whereby asylum seekers from the Balkan region can stay in the host country. This, in turn, implies that the expected duration of stay was substantial upon arrival in Germany, which might have fostered low-educated individuals to claim asylum there.^25^

Second, individuals could seek asylum with the aim of working in Germany, regardless of whether the job is in the formal or informal labor market. This motive could have been strengthened by origin-specific network ties that result from past (legal or illegal) migration to Germany. However, this potential channel is likely to be at play when the size of the network is relatively large. On the one hand, legal migration is proxied with the stock of valid residence permits at the end of the year in Germany (Eurostat 2017c). Among the five origin countries, 60% of the residence permits that were valid in 2013 (6% of all residence permits) were held by immigrants from Serbia. On the other hand, illegal migration is proxied through the evolution of the number of found illegal immigrants in Germany and the number of individuals who returned to their origin country after they received an order to leave. The number of undocumented immigrants from Afghanistan increased over time, but the reverse occurred for illegal immigrants from Iraq. Following the onset of the civil conflict, the number of illegal immigrants from Syria rose sharply.

**Fig. 3:**
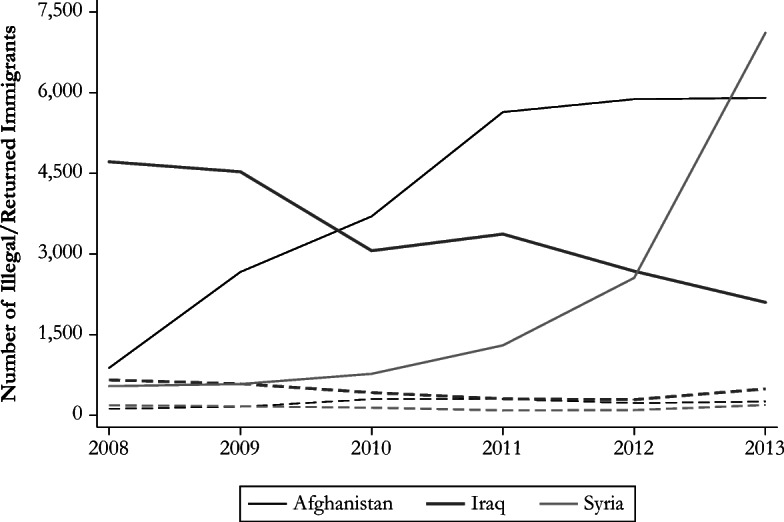
Illegal immigrants and returned individuals from conflict-affected countries. The solid lines represent illegal immigrants; the dashed lines correspond to returned individuals. *Source*: Author’s elaboration based on Eurostat (2017d, e).

**Fig. 4:**
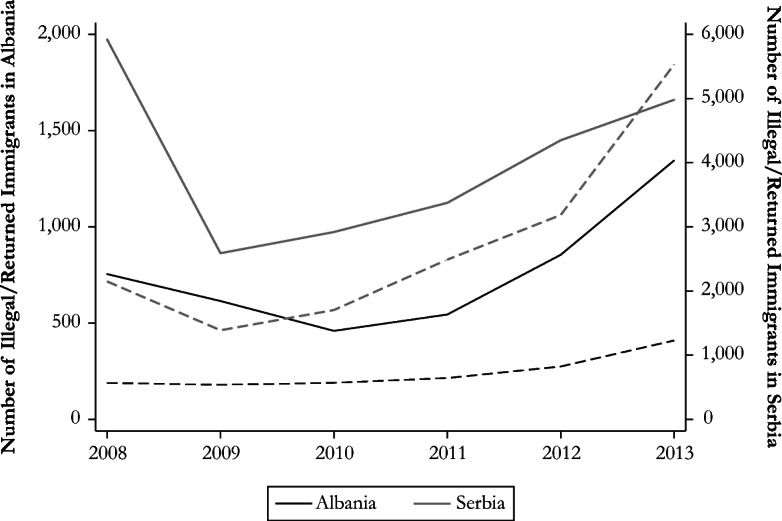
Illegal immigrants and returned individuals from Balkan countries. The solid lines represent illegal immigrants; the dashed lines correspond to returned individuals. The left (right) axis refers to figures for Albania (Serbia). *Source*: Author’s elaboration based on Eurostat (2017d, e).

Compared with the aforementioned figures, the number of deported immigrants is relatively constant and small, mainly because of the security conditions that prevail in the home country (Fig. 3). The two Balkan countries are strikingly different with respect to illegal migration (Fig. 4). The figures are, on average, small for Albania: 750 found illegal immigrants and 250 returned individuals. They are higher for Serbia and stand at, respectively, 4,000 and 2,700. The opportunity to come and stay illegally in Germany could have been enhanced by the lower migration costs faced to reach the host country from the Balkan region and by the fact that Albanians and Serbians can legally enter the Schengen area without a visa.

These descriptive statistics outline that past migration from Serbia to Germany and the related size of the network may have facilitated the arrival of Serbian asylum seekers and their entry into the German labor market. In addition, several stepwise changes in the German asylum policy improved conditions for accessing the labor market. The adjustments led to a reduction in waiting time to request a permit to work from 12 months to 9 months (from September 2013 until October 2014; Federal Ministry of Justice and Consumer Protection (Germany) 2013), and further to 3 months (from November 2014),^26^ provided that asylum seekers from the Balkan region were registered before September 2015; from this date onward, they were no longer allowed to work during the application processing time.^27^ In the IAB-BAMF-SOEP Refugee Sample, the last condition is fulfilled for most asylum seekers from Albania and Serbia (86%). At the end of the waiting period, compliance with various labor market regulations is assessed, so that asylum seekers can effectively be allowed to work in Germany. Altogether, Albanian and Serbian asylum seekers might have been attracted by the German labor market, but they would have encountered different hurdles when trying to find a job (at least, in the formal economy).

Finally, claiming refugee status directly benefits asylum seekers through the allocation of welfare provisions during the time required to review their application. The amounts depend on a range of characteristics, such as whether asylum seekers are living in public or private housing and the composition of one’s own family. For instance, if they are hosted in a government facility, two adult persons living in the same household as their partner each receive 129 euros per month, but the amount is 194 euros if they reside in a private dwelling (Federal Ministry of Justice and Consumer Protection (Germany) 2019). Given the low migration costs involved in migrating to Germany from the Balkan region and the longer time needed to evaluate the asylum claims, it might then have been economically worthwhile for Albanians and Serbians to seek asylum in Germany and receive welfare benefits until they were notified about their application.

## Conclusion

The distinction between asylum seekers and economic migrants is often made in the public debate based on the factors fostering the decision to migrate for each group. Unlike the determinants of economic migration, the drivers behind who is able to make her way to another country from the main asylum source countries have been rarely explored. The few studies that have focused on the self-selection of individuals in the context of forced migration are either related to past episodes of migration (Birgier et al. 2016) or based on data that are imperfectly representative of the origin population (Buber-Ennser et al. 2016) or of the asylum population at destination (Lange and Pfeiffer 2018). This study contributes to the literature through the use of individual-level and representative information for both asylum seekers in Germany and the population at origin. Specifically, the analysis is built on original data about asylum seekers in Germany complemented with surveys conducted in five key source countries, which offers an interesting variety of economic and security conditions at origin. The pattern of selection of asylum seekers from the origin population is examined with respect to education. The country-specific investigations provide evidence of positive selection on education for asylum seekers who fled Iraq and Syria, and shows mixed evidence for asylum seekers from Afghanistan. By contrast, individuals seeking asylum in Germany from Albania and Serbia are negatively selected relative to the home country population.

These patterns of selection on education are interpreted using differences in the expected duration of stay in Germany and in migration costs faced by asylum seekers when migrating to Germany. Specifically, I describe the decision of Albanians and Serbians to seek asylum in Germany (where their claims are almost certainly rejected) through the high expected processing time of their applications, which corresponds to the temporary legal period of stay in the host country. Lower expected duration of stay and migration costs may have triggered the observed negative selection on education of asylum seekers from the Balkan region, whereas a higher time horizon in Germany and migration costs may have driven the positive selection of asylum seekers from conflict-affected countries. Moreover, some network ties in Germany might have facilitated the arrival of asylum seekers from Serbia. This work suggests that the set of factors—especially the premigration socioeconomic status—influencing the decision of asylum seekers to migrate do not involve a sharp discontinuity with the determinants associated with migration decisions of economic migrants.

## Electronic supplementary material

ESM 1 (PDF 124 kb)

## Data Availability

The data sets analyzed during the current study are available from the author on reasonable request.

## Footnotes

1 The figure represents only a tiny fraction of all refugees. The number of refugees who were able to migrate to Germany is high compared with other European countries but small relative to neighboring countries of the main asylum source countries (UNHCR 2016). Thus, the observed pattern of selection is likely not to be representative of the entire population of forced migrants. See the Descriptive Evidence section for further discussion of this issue.

2 Hanson and McIntosh (2016) argued that the networks created by the surge of asylum seekers are likely to attract future migrants to come to Germany for years. The (short-lived) arrival of asylum seekers could then have a first-order impact on the long-term evolution of immigration to Germany, that the authors were otherwise predicting to decline.

3 Education is an important characteristic to evaluate the selection of asylum seekers. Specifically, the literature documents a positive correlation between education and wealth, and high-educated individuals are thus likely to be better-off (Card 1999). Because of data constraints, the analysis can account for only the level of education, which could be seen as a proxy of the socioeconomic condition of a given individual.

4 Throughout this article, Afghanistan, Iraq, and Syria are defined as *conflict-affected* countries. This denotation is straightforward for Syria because of the ongoing civil conflict, but is less so for Afghanistan and Iraq given that the conflict occurred in the past. The recent status in Europe of asylum seekers from the last two countries is different, mainly because of the conditions at origin. Some areas in Afghanistan are now considered safe, and the EU has signed an agreement (October 4, 2016) with Kabul (European Union (EU) and Islamic Republic of Afghanistan 2016) to implement the deportation of rejected asylum seekers from Afghanistan.

5 Unfortunately, I do not have information about the ethnicity of asylum seekers because German survey questions (mostly for historical reasons) do not ask about it. Thus, I cannot investigate whether asylum seekers from Albania and Serbia are predominantly Sinti or Roma, a group that is heavily stigmatized and partly persecuted in the Balkan countries. Moreover, this would also have been an additional explanation for the negative selection of asylum seekers from the Balkan region given that Sinti and Roma often do not participate in the general educational system.

6 In 2015, the acceptance rate was 72.8% for asylum applications from Afghanistan, 98.3% for Iraq, and 97.7% for Syria. Only 0.2% (0.1%) of Albanian (Serbian) asylum seekers were granted a refugee status (Eurostat 2018).

7 The decision to seek refuge in Germany from Albania has been related to the influence of smugglers along refugee routes toward European countries, with asylum seekers grossly overestimating the chances of being granted a refugee status. The German Embassy in Tirana implemented ad campaigns to warn people against seeking asylum in Germany (Meisner 2015).

8 The origin-specific expected processing time of the asylum applications averaged at 9 and 15.5 months for, respectively, Albania and Serbia in 2015 (see section on empirical results).

9 The computation of the returns to education at origin and at destination would be helpful to predict the selection patterns. Unfortunately, such a computation is not possible with the available data, thus preventing a more structural estimation by first estimating income and then estimating the relationship between location choices and income, as in Bertoli et al. (2013).

10 Recently, Chen et al. (2017) studied the self-selection of Pakistani migrants who decided *ex ante* between temporary and permanent internal migration. The authors demonstrated that the expected duration of stay affects the selection of migrants and that a permanent move drives a positive pattern of selection with respect to education.

11 The *safe country of origin* concept is a presumption that certain countries can be designated as safe for their nationals to the extent that “it can be shown that there is generally and consistently no persecution as defined in Article 9 of Directive 2004/83/EC, no torture or inhuman or degrading treatment or punishment and no threat by reason of indiscriminate violence in situations of international or internal armed conflict” (Council of the European Union 2005). This implies that a claim for international protection by an applicant from a safe country of origin is likely to be considered unfounded.

12 Law on classification of further states as safe countries of origin and on the facilitation of access to the labor market for asylum seekers and tolerated foreigners (Federal Ministry of Justice and Consumer Protection (Germany) 2014).

13 Law for an acceleration of asylum procedures (Federal Ministry of Justice and Consumer Protection (Germany) 2015).

14 The time lapse between the data collection and the asylum arrival implies that the analysis is not directly comparing asylum seekers with individuals who stayed in the source country but instead with the entire population at origin, thus diluting any pattern of self-selection.

15 Considering Syria, the Central Microdata Catalog of the World Bank clearly indicates that the sample is “nationally representative and cover the whole of Syria” (UNICEF 2006).

16 The number of cases presented in Table 2 differs from the one reported in Table 1 because of sample restrictions on the age of individuals and from missing values with respect to the level of education (Table A2.1.1, online appendix).

17 Using the IAB-SOEP Migration Sample (2015), I compare the education profile of Balkan asylum seekers with the one of economic migrants, who migrated to Germany mainly through family reunification. The selection pattern with respect to education of Serbian migrants is close to the one of the origin population, whereas economic migrants from Albania seem to be selected from the middle of the education distribution at origin. The statistics are available from the author upon request.

18 Information used to build some control variables relates to the premigration period. Section A5 of the online appendix demonstrates that the retrospective language proficiency in German of asylum seekers is not correlated with the time they have spent in Germany.

19 I do not have individual-level information on the perceived level of insecurity in Syria, which I circumvent by matching the sample with aggregate data from the Political Terror Scale (PTS 2017) and the Freedom House (FH 2017) based on the date of departure for asylum seekers and on the survey date for the origin population. The latter implies values of PTS (3, from the U.S. State Department) and FH (6, for Civil Liberties) indices that clearly do not represent the situation in Syria after 2011. I solve the issue by randomly assigning a year (between 2006 and 2016) to each individual in the MICS sample and use it as the base year to merge with the aggregate variables. An overview of the outcome of this procedure is given in Table A1.3 in the online appendix.

20 The median cost of migration and the median time to reach Germany come directly from the IAB-BAMF-SOEP Refugee Sample (2017). Information about the latter is obtained from answers to the following question: “How many days did it take to travel from your country of birth to Germany?”

21 Voluntary returns from Germany are implemented via the REAG/GARP programme run by the International Organisation for Migration. In 2016, Albania (31.3%) and Serbia (11.4%) were the two most important countries of origin involved in voluntary returns (European Migration Network/Federal Office for Migration and Refugees (EMN/BAMF) 2016:60, figure 5).

22 Recently, Borjas (2017) documented that the rate of returns to schooling (i.e., the coefficient of years of schooling in the log wage regression) is lower for undocumented immigrants in the United States than for legal immigrants or native workers.

23 In 2015, the Federal Office for Migration and Refugees (BAMF) took on average 5.2 months to determine whether the request would be accepted; the waiting period was 7.1 months in 2016, increasing to 8.1 months in the fourth quarter of 2016 (Federal Government (Germany) 2017).

24 I calculate the expected processing time by comparing the number of origin-specific pending applications at the end of month (*t* – 1) with the number of months (from 1 to 36), over which it is necessary to cumulate applications (from *t* – 1) to reach the number of pending applications. I then take the average of the generated variable for each year and each origin country (Eurostat 2017a, b).

25 In principle, I could use the date of arrival in Germany to check whether asylum seekers from Balkan countries, who arrived when the time to process the applications and the recognition rate were high, are less negatively selected than those who arrived when the decision time and acceptance rates were lower. However, the small number of observations prevents an econometric analysis of the mechanism. Descriptive evidence supporting the aforementioned assumption is available from the author upon request.

26 Law for the classification of additional states as safe countries of origin and to facilitate the access to the labor market for asylum seekers and tolerated foreigners (Federal Ministry of Justice and Consumer Protection (Germany) 2014).

27 Law for the acceleration of asylum procedures (Federal Ministry of Justice and Consumer Protection (Germany) 2015).
