# Supplementary material for: Self-selection of Asylum Seekers: Evidence From Germany
Source: Demography. 2020 May 4;57(3):1089–116. doi: 10.1007/s13524-020-00873-9 (PMC7329792; doi:10.1007/s13524-020-00873-9)
Supplement: Supplementary file 1 — (PDF 124 kb) [file 13524_2020_873_MOESM1_ESM.pdf]

Online Appendix

A1 Statistics of origin-specific surveys

Table A1.1: Number of cases for Afghanistan by wave, gender and age

	Men		Women		
Wave	Up to 30	Over 30	Up to 30	Over 30	Total
2011	1,475	2,028	1,309	1,344	6,156
	(12.7)	(13.6)	(10.8)	(11.3)	(12.2)
2012	2,046	2,643	1,570	1,484	7,743
	(17.6)	(17.7)	(13.0)	(12.5)	(15.3)
2013	2,230	2,896	1,509	1,592	8,227
	(19.2)	(19.4)	(12.5)	(13.4)	(16.3)
2014	1,701	2,219	2,376	2,203	8,499
	(14.6)	(14.8)	(19.7)	(18.5)	(16.8)
2015	1,811	2,205	2,345	2,342	8,703
	(15.6)	(14.7)	(19.3)	(19.7)	(17.2)
2016	2,364	2,970	2,970	2,953	11,257
	(20.3)	(19.9)	(24.6)	(24.8)	(22.3)
Total	11,627	14,961	12,079	11,918	50,585
	[23.0]	[29.6]	[23.9]	[23.6]	(100.0)

Notes: Percentages by column are displayed in parentheses, whereas shares by line are given in brackets. Individuals aged below 18 and above 64 are not included.
Source: Author's elaboration based on SAP (2011-16).

Table A1.2: Number of cases for Iraq by year, gender and age

	Men		Women		
Year	Up to 30	Over 30	Up to 30	Over 30	Total
2012	19,800	23,005	18,772	23,020	84,597
	(96.1)	(95.9)	(96.3)	(96.1)	(96.1)
2013	798	975	724	946	3,443
	(3.9)	(4.1)	(3.7)	(4.0)	(3.9)
Total	20,598	23,980	19,496	23,966	88,040
	[23.4]	[27.2]	[22.1]	[27.2]	(100.0)

Notes: Percentages by column are displayed in parentheses, whereas shares by line are given in brackets. Individuals aged below 18 and above 64 are not included.
Source: Author's elaboration based on HSES (2012-13).

Table A1.3: Adjustments of PTS and FH indices

	2006	2007	2008	2009	2010	2011	2012	2013	2014	2015	2016	Mean ∣ Total
PTS (US State Department)	3	3	4	4	4	5	5	5	5	5	5	4.36
FH (Civil Liberties)	6	6	6	6	6	6	7	7	7	7	7	6.55
Number of cases	5,142	4,931	4,918	5,023	5,070	5,005	4,990	5,079	5,090	5,023	5,006	55,277

Note: Number of cases corresponds to individuals in MICS (2006).
Source: Author's elaboration based on Gibney et al. (2017) and Freedom House (2017), Civil Liberties.

Table A1.4: Number of cases for Serbia by wave, gender and age

	Men		Women		
Wave	Up to 30	Over 30	Up to 30	Over 30	Total
2013	1,614	4,778	1,505	4,982	12,879
	(34.1)	(34.9)	(34.6)	(35.4)	(35.0)
2014	1,598	4,546	1,448	4,662	12,254
	(33.7)	(33.2)	(33.3)	(33.2)	(33.3)
2015	1,524	4,361	1,394	4,411	11,690
	(32.2)	(31.9)	(32.1)	(31.4)	(31.7)
Total	4,736	13,685	4,347	14,055	36,823
	[12.9]	[37.2]	[11.8]	[38.2]	(100.0)

Notes: Percentages by column are displayed in parentheses, whereas shares by line are given in brackets. Individuals aged below 18 and above 64 are not included.
Source: Author's elaboration based on EU-SILC (2013-15).

A2 Data harmonisation

A2.1 Level of education

The empirical analysis focuses on the selection of asylum seekers from the origin population with respect to education[1]. Two steps are implemented to combine available information on the educational attainment of individuals[2]. First, the answers about the level of education in each questionnaire are divided into six different categories, i.e. no formal education, primary, lower secondary, upper secondary education, vocational training and university. Tables A2.1.1 and A2.1.2 detail the procedure that has been followed to assign answers about education to each group for the asylum seekers and the origin population, respectively.

[1] When discussing about the level of education of asylum seekers hosted by Germany, we only consider the education obtained in the origin country. The paper does not account for further education which might have been attended in the host country.
[2] The analysis does not include individuals, who were attending education in the origin country at the time the surveys were carried out.

Nevertheless, the six categories are not included in every survey, e.g. studies carried out in Afghanistan and Syria do not contain information on vocational training. Therefore, the initial binary indicators are grouped, so that the final variable of interest is composed of three levels: (*i*) Primary education or less, which refers to cases without education and with primary education (*ii*) Secondary education, which contains individuals who attended lower, upper secondary education and vocational training and (*iii*) Tertiary education, which encloses those who went to university.

Table A2.1.1: Level of education of asylum seekers

	Question	Answers	Frequency	Code
School certificate	In which year did you last attended a general school?	I did not attend school	13.11	A
	Have you attended school in another country than Germany?	No		A
	With what kind of graduation you finished school there?	Left school with no qualifications	23.57	B
		Middle school leaving certificate	20.87	C
		Practical-based further education certificate	8.08	D
		General-based further education certificate	23.44	E
		Certificate from a different school	2.47	F
		No information	8.46	
Vocational training	Were you in a country other than Germany in a vocational training or have you studied in another country than Germany?	No	74.90	
	What kind of vocational training was that?	(Long) training in a company		
	How did you finish this training?	Aborted prematurely	0.33	1
		Completed without certificate	0.93	2
		Completed with certificate	2.73	3
		Attended a vocational school		
		Aborted prematurely	0.19	4
		Completed without certificate	0.21	5
		Completed with certificate	1.83	6
		Other training		
		Aborted prematurely	0.06	7
		Completed without certificate	0.31	8
		Completed with certificate	0.48	9
		University with practical or theoretical orientation visited / Graduate degree		
		Aborted prematurely	4.49	10
		Completed without certificate	0.99	11
		Completed with certificate	10.69	12
		No information	1.86	
Level of education		No education	36.45	A,B
		Primary education	17.24	C
		Lower secondary	17.66	(D,E,F) + (1,2,4,5,7,8)
		Upper secondary	5.04	(C,D,E,F) + (10,11)
		Vocational	5.04	(3,6,9)
		University	10.69	12
		No information	7.87	

Notes: Statistics reported in the fourth column are associated to the full sample of asylum seekers and are therefore not representative of the five selected origin countries. Figures are weighted to be representative of the (recent) asylum population in Germany.
Source: Author's elaboration based on IAB-BAMF-SOEP Refugee Sample (2017).

Table A2.1.2: Level of education in the origin country

Origin	Question	Answers	Frequency	Categories
Afghanistan	What is the highest level of school you completed?	Never went to school	55.87	No education
		Islamic education at Madrassa	0.52	No education
		Informal schooling at home or at a literacy class	1.88	No education
		Primary school, incomplete (classes 1 to 5)	8.22	Primary
		Primary school, complete (finished class 6)	4.71	Primary
		Secondary education, incomplete (classes 7 to 8)	4.73	Primary
		Secondary education, complete (finished class 9)	2.81	Lower secondary
		High school (classes 10 to 12)	5.05	Lower secondary
		High school incomplete (classes 10-11)	2.58	Lower secondary
		High school complete (finished class 12)	7.79	Upper secondary
		14th grade incomplete (class 13)	0.66	Upper secondary
		14th grade complete (finished class 14)	2.18	Upper secondary
		University education incomplete	0.54	University
		University education or above	2.42	University
		No information	0.04	
Iraq	What was the highest certificate you attained?	No certificate / Never went to school	40.04	No education
		Elementary	25.31	Primary
		Intermediate	8.39	Lower secondary
		Basic	0.37	Lower secondary
		Secondary	3.95	Upper secondary
		Diploma from an institution	6.00	Upper secondary
		Vocational	1.23	Vocational
		Bachelor degree	5.88	University
		Higher diploma	0.09	University
		Master degree	0.23	University
		PhD (Doctorate)	0.10	University
		No information	8.41	
Syria	What is the highest level of school you attended?	Pre-school / Never attended school	19.66	No education
		Primary	32.14	Primary
		Preparatory	21.04	Lower secondary
		Secondary	10.51	Upper secondary
		Post secondary institute	5.83	Upper secondary
		University and higher	5.18	University
		No information	5.64	
Albania	What is the highest grade you have completed in school?	None	2.73	No education
		8 or 9 years school	44.46	Primary
		Technicum < 2 years	0.71	Lower secondary
		Gymnasium (secondary general)	27.41	Upper secondary
		Vocational 2-3 years	1.94	Vocational
		Vocational 4/5 years	2.80	Vocational
		University - Albania	12.25	University
		University - Abroad	0.15	University
		Master - Albania	0.68	University
		Master - Abroad	0.02	University
		Doctorate/PhD - Albania	0.08	University
		Doctorate/PhD - Abroad	0.01	University
		No information	6.77	
Serbia	Highest ISCED level attained	No education	1.11	No education
		Pre-primary education	2.04	No education
		Primary education	7.60	Primary
		Lower secondary education	16.50	Lower secondary
		Upper secondary education	45.49	Upper secondary
		Post-secondary non tertiary education	1.42	Vocational
		First and second stage of tertiary education	16.72	University
		No information	9.12	

Notes: For Syria, an individual is assigned to one category if at least one grade has been completed at a given level of education, otherwise the level immediately below is attributed. For Serbia, the original questionnaire (2013) asked the acquired education level, with the following answers: (*i*) No school, (*ii*) 4th grade of primary school, (*iii*) 5th-8th grade of primary school, (*iv*) Primary school, (*v*) 3-year secondary school, (*vi*) 4-year secondary school, (*vii*) Specialisation after secondary school, (*viii*) College - 1st faculty degree, (*ix*) Higher education and (*x*) Doctoral studies. However, figures related to this classification are not accessible. Source: Author's elaboration based on the full sample (i.e., without restriction on individuals' age) of the following datasets: SAP (2011-16), HSES (2012-13), MICS (2006), LSMS (2012) and EU-SILC (2013-15).

A2.2 Insecurity in the home country

The subjective perceptions on insecurity (labelled *Insecurity*) in the origin site are reported in three samples (Afghanistan, Iraq and Serbia). The starting point to match information among the different data sources is the answers collected in the IAB-BAMF-SOEP Refugee Sample. Asylum seekers were asked the following semi-open query: "What were the reasons for leaving your country of origin?" and, among all propositions, four of them are retained to build a binary variable indicating whether people felt threatened (at least, by one of the selected propositions) before the migration to Germany. The possible answers are: (*i*) "Fear or violent conflicts or war", (*ii*) "Fear or forced recruitment by military or armed groups", (*iii*) "Persecution" and (*iv*) "Discrimination (ethnic, religious, etc.)".

Table A2.2: Subjective perceptions of insecurity in the origin country

Country	Question	Available answers
Afghanistan	How often do you fear for your own personal safety or security or for that of your family these days?	Never, rarely → Insecurity = 0<br>Sometimes, often, always → Insecurity = 1
Iraq	In general, how satisfied or unsatisfied are you with you local security level?	Very satisfied → Insecurity = 0<br>Fairly, not very, not at all satisfied → Insecurity = 1
Serbia (only 2013)	How safe do you feel in the area where you live?	Very safe → Insecurity = 0<br>Mostly, not feel quite safe, not feel safe at all → Insecurity = 1

Source: Author's elaboration based on SAP (2011-16), HSES (2012-13) and EU-SILC (2013).

Then, the variable is combined with relevant individual characteristics encompassed in the three origin-specific data sets. For each case, a binary indicator has been derived from the ordinal answers that were available for the respondents. Table A2.2 presents the question about insecurity and the procedure followed to assign the replies to to the variable.

A3 Definition of the variables

Table A3: Definition of the variables

Variable	Definition	Additional comments
Age	Age at the time of the survey (18 to 64)	Include age squared in the regressions
Education*	Level of education. Divided into 3 binary categories: primary or less, secondary and tertiary education	
Speaks German*	1 if an individual is able to speak German, 0 otherwise	Available only for Afghanistan and Albania
Male	1 if an individual is a male, 0 otherwise	
Married	1 if an individual is married, 0 otherwise	Not available for Syria
Insecurity*	1 if an individual feels unsafe in the origin country, 0 otherwise	Not available for Syria and Albania. Replaced by PTS and FH indices for Syria
Religion	Religious affiliation. Divided into 4 binary categories: Atheist, Muslim, Orthodox/Other and Catholic	Available only for Albania
Occupational status*	Divided into 4 dummies: never worked, worker, self-employed and employee	Available only for Serbia

Notes: Variables with a star superscript denote characteristics of asylum seekers that refer to the pre-migration period. For instance, the occupational status corresponds to the position held before they left the origin country.

A4 List of variables and summary statistics

Table A4.1: List of variables and summary statistics - Afghanistan

Mig	Variable	Mean	SD	N	Mig	Variable	Mean	SD	N
	**Level of education**					**Level of education**			
	Primary or less	0.76	0.43	50,406		Primary or less	0.70	0.46	442
	Secondary	0.21	0.41	50,406		Secondary	0.26	0.44	442
	Tertiary	0.03	0.17	50,406		Tertiary	0.04	0.20	442
0	Age	33.70	11.59	50,406	1	Age	27.93	8.79	442
	Speaks German	0.00	0.03	50,406		Speaks German	0.05	0.22	442
	Male	0.52	0.50	50,406		Male	0.77	0.42	442
	Married	0.79	0.40	50,406		Married	0.40	0.49	442
	Insecurity	0.63	0.48	50,406		Insecurity	0.94	0.24	442

Notes: Mig refers to the migration status. It takes the value 0 for the origin population and 1 for asylum seekers.
Source: Author's elaboration based on SAP (2011-16) and IAB-BAMP-SOEP Refugee Sample (2017).

Table A4.2: List of variables and summary statistics - Iraq

Mig	Variable	Mean	SD	N	Mig	Variable	Mean	SD	N
	**Level of education**					**Level of education**			
	Primary or less	0.70	0.46	80,722		Primary or less	0.68	0.47	485
	Secondary	0.23	0.42	80,722		Secondary	0.20	0.40	485
	Tertiary	0.07	0.26	80,722		Tertiary	0.12	0.33	485
0	Age	35.88	12.21	80,722	1	Age	30.74	9.54	485
	Male	0.49	0.50	80,722		Male	0.73	0.44	485
	Married	0.74	0.44	80,722		Married	0.50	0.50	485
	Insecurity	0.60	0.49	80,722		Insecurity	0.98	0.14	485

Notes: Mig refers to the migration status. It takes the value 0 for the origin population and 1 for asylum seekers.
Source: Author's elaboration based on HSES (2012-13) and IAB-BAMP-SOEP Refugee Sample (2017).

Table A4.3: List of variables and summary statistics - Syria

Mig	Variable	Mean	SD	N	Mig	Variable	Mean	SD	N
	**Level of education**					**Level of education**			
	Primary or less	0.52	0.50	51,968		Primary or less	0.47	0.50	2,046
	Secondary	0.42	0.49	51,968		Secondary	0.36	0.48	2,046
	Tertiary	0.06	0.23	51,968		Tertiary	0.16	0.37	2,046
0	Age	35.28	12.50	51,968	1	Age	31.11	10.31	2,046
	Male	0.51	0.50	51,968		Male	0.74	0.44	2,046
	PTS	4.36	0.77	51,968		PTS	4.97	0.23	2,046
	FH CL	6.55	0.50	51,968		FH CL	6.99	0.08	2,046

Notes: Mig refers to the migration status. It takes the value 0 for the origin population and 1 for asylum seekers. PTS corresponds to Political Terror Scale, while FH CL is the Civil Liberties index from Freedom House.
Source: Author's elaboration based on MICS (2006), Gibney et al. (2017), Freedom House (2017) and IAB-BAMP-SOEP Refugee Sample (2017).

Table A4.4: List of variables and summary statistics - Albania

Mig	Variable	Mean	SD	N	Mig	Variable	Mean	SD	N
	**Level of education**					**Level of education**			
	Primary or less	0.46	0.50	14,829		Primary or less	0.48	0.50	46
	Secondary	0.38	0.49	14,829		Secondary	0.52	0.51	46
	Tertiary	0.16	0.36	14,829		Tertiary	0.00	0.05	46
	Age	41.23	13.14	14,829		Age	30.23	10.09	46
	Speaks German	0.01	0.08	14,829		Speaks German	0.20	0.40	46
0	Male	0.50	0.50	14,829	1	Male	0.72	0.45	46
	Married	0.76	0.43	14,829		Married	0.42	0.50	46
	**Religion**					**Religion**			
	Atheist	0.01	0.08	14,829		Atheist	0.02	0.13	46
	Muslim	0.82	0.38	14,829		Muslim	0.86	0.35	46
	Orthodox/Other	0.08	0.27	14,829		Orthodox/Other	0.03	0.18	46
	Catholic	0.10	0.29	14,829		Catholic	0.09	0.29	46

Notes: Mig refers to the migration status. It takes the value 0 for the origin population and 1 for asylum seekers.
Source: Author's elaboration based on LSMS (2012) and IAB-BAMP-SOEP Refugee Sample (2017).

Table A4.5: List of variables and summary statistics - Serbia

Mig	Variable	Mean	SD	N	Mig	Variable	Mean	SD	N
	**Level of education**					**Level of education**			
	Primary or less	0.04	0.20	33,395		Primary or less	0.81	0.39	43
	Secondary	0.76	0.43	33,395		Secondary	0.19	0.39	43
	Tertiary	0.20	0.40	33,395		Tertiary			43
	Age	44.07	12.57	33,395		Age	28.24	7.40	43
	Male	0.50	0.50	33,395		Male	0.72	0.46	43
0	Married	0.61	0.49	33,395	1	Married	0.58	0.50	43
	**Occupation**					**Occupation**			
	Never worked	0.15	0.35	33,395		Never worked	0.53	0.50	43
	Worker	0.03	0.17	33,395		Worker	0.40	0.50	43
	Self-employed	0.13	0.34	33,395		Self-employed	0.05	0.21	43
	Employee	0.69	0.46	33,395		Employee	0.02	0.13	43
	Insecurity	0.60	0.49	8,358		Insecurity	0.62	0.49	42

Notes: Mig refers to the migration status. It takes the value 0 for the origin population and 1 for asylum seekers.
Source: Author's elaboration based on EU-SILC (2013-15) and IAB-BAMP-SOEP Refugee Sample (2017).

A5 Consistency of language proficiency

This section addresses a potential concern associated to retrospective (i.e., linked to the pre-migration period) questions that were asked in the Refugee Sample. For instance, the ability to speak German is defined from the following question: "How well could you speak the German language before you move

to Germany?". An issue will arise if the answers are a function of the time spent in Germany, so that they will be contaminated by the current language aptitude of asylum seekers. To evaluate whether this problem might exists in the empirical investigation, the relevant variable is regressed on the years and months since arrival in Germany. The results obtained using the linear probability model are given in Table A5.

Table A5: Language proficiency and time spent in Germany

Dependent variable: 1 if an individual is able to speak German before migration, 0 otherwise						
	All		Afghanistan		Albania	
Years since arrival	0.003		−0.007		−0.025	
	(0.004)		(0.011)		(0.035)	
Months since arrival		0.000		−0.001		−0.006
		(0.000)		(0.001)		(0.004)
Observations	3,326	3,229	521	499	52	50

Notes: All models are estimated using OLS. Robust standard errors in parentheses. ***, **, and * denote significance at the 1, 5, and 10 percent levels, respectively. All represents the five selected origin countries.
Source: Author's elaboration based on SAP (2011-16), LSMS (2012) and IAB-BAMF-SOEP Refugee Sample (2017).

All coefficients are not significant, regardless of the time variable taken into account. This outcome supports the idea that the time spent in the host country is likely not to influence the estimates of the language variable included in the specifications associated to Afghanistan and Albania.

A6 Results of the logistic regressions

Table A6.1: Self-selection of asylum seekers from conflict-affected countries

Dependent variable: 1 if an individual has migrated, 0 otherwise
Benchmark group: Tertiary education

	Afghanistan			Iraq		Syria		
	(1)	(2)	(3)	(4)	(5)	(6)	(7)	(8)
**Level of Education**								
Primary or less	-0.626***	-0.719***	-0.434*	-0.688***	-0.914***	-0.838***	-0.841***	-0.842***
	(0.216)	(0.215)	(0.255)	(0.152)	(0.154)	(0.068)	(0.071)	(0.071)
Secondary	-0.984***	-1.037***	-0.794***	-0.730***	-0.844***	-1.316***	-1.326***	-1.329***
	(0.226)	(0.228)	(0.267)	(0.177)	(0.178)	(0.071)	(0.074)	(0.074)
Age	0.213***	0.194***	0.199***	0.125***	0.127***	0.165***	0.166***	0.166***
	(0.040)	(0.039)	(0.039)	(0.027)	(0.028)	(0.015)	(0.015)	(0.015)
Age^2	-0.003***	-0.003***	-0.003***	-0.002***	-0.002***	-0.002***	-0.002***	-0.002***
	(0.001)	(0.001)	(0.001)	(0.000)	(0.000)	(0.000)	(0.000)	(0.000)
Male	0.477***	0.554***	0.531***	0.494***	0.553***	0.553***	0.560***	0.560***
	(0.107)	(0.107)	(0.108)	(0.096)	(0.096)	(0.048)	(0.049)	(0.049)
Married	-1.109***	-1.091***	-1.086***	-0.423***	-0.437***			
	(0.133)	(0.129)	(0.130)	(0.103)	(0.103)			
Insecurity		2.162***	2.161***		3.811***			
		(0.191)	(0.191)		(0.337)			
Speaks German		4.506***						
			(0.406)					
PTS							2.700***	
							(0.146)	
FH CL								4.493***
								(0.231)
Observations	50,848	50,848	50,848	81,207	81,207	54,014	54,014	54,014
McFadden's R^2	0.028	0.074	0.097	0.018	0.103	0.038	0.146	0.164

Notes: All models are estimated using logistic regressions and the reported coefficients are in log-odds units. McFadden's $R^2 = 1 - ln(L_M)/ln(L_0)$, with L_M, the likelihood of the estimated model and L_0, the likelihood of the model without predictors. Robust standard errors in parentheses. ***, **, and * denote significance at the 1, 5, and 10 percent levels, respectively. PTS corresponds to Political Terror Scale, while FH CL is the Civil Liberties index from Freedom House.

Source: Author's elaboration based on SAP (2011-16), HSES (2012-13), MICS (2006), Gibney et al. (2017), Freedom House (2017) and IAB-BAMP-SOEP Refugee Sample (2017).

Table A6.2: Self-selection of asylum seekers from Balkan countries

Dependent variable: 1 if an individual has migrated, 0 otherwise
Benchmark group: Tertiary education (Albania), Secondary or more (Serbia)

	Albania			Serbia		
	(1)	(2)	(3)	(4)	(5)	(6)
**Level of education**						
Primary or less	2.180**	2.553**	2.277**	5.357***	4.699***	5.382***
	(1.004)	(1.071)	(0.985)	(0.402)	(0.445)	(0.464)
Secondary	2.057**	2.355**	2.066**			
	(1.016)	(1.056)	(1.012)			
Age	0.160	0.160	0.158	0.236**	0.226*	0.178
	(0.122)	(0.121)	(0.120)	(0.112)	(0.118)	(0.127)
Age^2	-0.003*	-0.003*	-0.003*	-0.005***	-0.005***	-0.004**
	(0.002)	(0.002)	(0.002)	(0.002)	(0.002)	(0.002)
Male	-0.359	-0.400	-0.383	0.609*	0.662**	1.009**
	(0.306)	(0.305)	(0.307)	(0.344)	(0.334)	(0.406)
Married	0.727	0.778	0.762	1.346***	1.415***	1.814***
	(0.533)	(0.531)	(0.528)	(0.369)	(0.356)	(0.487)
Speaks German		3.122***				
		(0.664)				
**Religion**						
Atheist			2.441***			
			(0.554)			
Orthodox / Other			0.366			
			(0.619)			
Catholic			0.399			
			(0.422)			
**Occupation**						
No work					2.740***	
					(0.998)	
Worker					4.802***	
					(1.016)	
Self-employed					2.044	
					(1.254)	
Insecurity						-0.335
						(0.374)
Observations	14,875	14,875	14,875	33,438	33,438	8,400
McFadden's R^2	0.054	0.074	0.073	0.435	0.515	0.518

Notes: All models are estimated using logistic regressions. McFadden's $R^2 = 1 - ln(L_M)/ln(L_0)$, with L_M, the likelihood of the estimated model and L_0, the likelihood of the model without predictors. Robust standard errors in parentheses. ***, **, and * denote significance at the 1, 5, and 10 percent levels, respectively. Muslim is the benchmark category to analyse the religious affiliation of Albanians. Employee (both with and without supervision tasks) is the reference group to interpret the occupational status in Serbia. Information about insecurity in Serbia is only available in the 2013 wave, which explains the number of observations reported in column (6).
Source: Author's elaboration based on LSMS (2012), EU-SILC (2013-15) and IAB-BAMF-SOEP Refugee Sample (2017).

A7 Robustness checks

Table A7.1: Selection of asylum seekers through the analysis of rare events

Dependent variable: 1 if an individual has migrated, 0 otherwise
Benchmark group: Tertiary education, Secondary or more (Serbia)

	Afghanistan			Iraq		Syria		
	(1)	(2)	(3)	(4)	(5)	(6)	(7)	(8)
Primary or less	−0.008**	−0.010***	−0.005*	−0.006***	−0.008***	−0.049***	−0.046***	−0.045***
	(0.003)	(0.004)	(0.003)	(0.002)	(0.002)	(0.005)	(0.005)	(0.005)
Secondary	−0.011***	−0.012***	−0.008**	−0.006***	−0.008***	−0.064***	−0.061***	−0.061***
	(0.003)	(0.004)	(0.003)	(0.002)	(0.002)	(0.005)	(0.005)	(0.005)
McFadden's R^2	0.028	0.075	0.098	0.018	0.103	0.038	0.146	0.164
Observations	50,848	50,848	50,848	81,207	81,207	54,014	54,014	54,014

	Albania			Serbia		
	(10)	(11)	(12)	(13)	(14)	(15)
Primary or less	0.003***	0.004***	0.004***	0.047***	0.022***	0.129***
	(0.001)	(0.001)	(0.001)	(0.008)	(0.004)	(0.019)
Secondary	0.003***	0.003***	0.003***			
	(0.001)	(0.001)	(0.001)			
McFadden's R^2	0.053	0.077	0.078	0.455	0.540	0.549
Observations	14,875	14,875	14,875	33,438	33,438	8,400

Notes: All models are estimated using penalized logistic regressions and the reported coefficients are the average marginal effects. McFadden's $R^2 = 1 - ln(L_M)/ln(L_0)$ with L_M, the likelihood of the estimated model and L_0, the likelihood of the model without predictors. Robust standard errors in parentheses. ***, **, and * denote significance at the 1, 5, and 10 percent levels, respectively.
Source: Author's elaboration based on SAP (2011-16), HSES (2012-13), MICS (2006), LSMS (2012), EU-SILC (2013-15) and IAB-BAMF-SOEP Refugee Sample (2017).

Table A7.2: Selection with respect to urban/rural origin population

Dependent variable: 1 if an individual has migrated, 0 otherwise
Benchmark group: Tertiary education, Secondary or more (Serbia)

	Afghanistan			Iraq		Syria		
	(1)	(2)	(3)	(4)	(5)	(6)	(7)	(8)
Primary or less	0.030**	0.024	0.037***	−0.004*	−0.008***	−0.041***	−0.037***	−0.037***
	(0.015)	(0.015)	(0.014)	(0.002)	(0.003)	(0.007)	(0.006)	(0.006)
Secondary	−0.037***	−0.038***	−0.026**	−0.007***	−0.010***	−0.079***	−0.074***	−0.074***
	(0.013)	(0.014)	(0.013)	(0.002)	(0.003)	(0.007)	(0.006)	(0.006)
McFadden's R^2	0.061	0.115	0.134	0.019	0.113	0.042	0.164	0.184
Observations	6,515	6,515	6,515	45,972	45,972	30,736	30,736	30,736

	Albania			Serbia	
	(10)	(11)	(12)	(13)	(14)
Primary or less	0.004**	0.005***	0.005***	0.124***	0.062***
	(0.002)	(0.002)	(0.002)	(0.018)	(0.010)
Secondary	0.006**	0.007***	0.006***		
	(0.003)	(0.002)	(0.002)		
McFadden's R^2	0.048	0.075	0.093	0.500	0.566
Observations	7,277	7,277	7,277	9,240	9,240

Notes: All models are estimated using logistic regressions and the reported coefficients are the average marginal effects. McFadden's $R^2 = 1 - ln(L_M)/ln(L_0)$ with L_M, the likelihood of the estimated model and L_0, the likelihood of the model without predictors. Robust standard errors in parentheses. ***, **, and * denote significance at the 1, 5, and 10 percent levels, respectively. Due to data limitations, column (6) of Table 8 can not be replicated.

Source: Author's elaboration based on SAP (2011-16), HSES (2012-13), MICS (2006), LSMS (2012), EU-SILC (2013-15) and IAB-BAMF-SOEP Refugee Sample (2017).

Table A7.3: Selection on education of male asylum seekers

Dependent variable: 1 if an individual has migrated, 0 otherwise
Benchmark group: Tertiary education

	Afghanistan			Iraq		Syria		
	(1)	(2)	(3)	(4)	(5)	(6)	(7)	(8)
Primary or less	−0.003	0.001	−0.005	−0.006***	−0.008***	−0.043***	−0.039***	−0.039***
	(0.004)	(0.003)	(0.004)	(0.002)	(0.003)	(0.007)	(0.006)	(0.006)
Secondary	−0.009**	−0.005*	−0.011***	−0.005**	−0.007***	−0.068***	−0.063***	−0.063***
	(0.004)	(0.003)	(0.004)	(0.002)	(0.003)	(0.007)	(0.006)	(0.006)
McFadden's R^2	0.038	0.066	0.095	0.021	0.118	0.031	0.139	0.163
Observations	26,781	26,781	26,781	40,337	40,337	27,746	27,746	27,746

Benchmark group: Secondary education or more

	Albania			Serbia		
	(10)	(11)	(12)	(13)	(14)	(15)
Primary or less	0.002*	0.003*	0.002*	0.054***	0.024***	0.156***
	(0.001)	(0.002)	(0.001)	(0.012)	(0.005)	(0.034)
McFadden's R^2	0.027	0.062	0.034	0.487	0.626	0.595
Observations	7,524	7,524	7,524	16,815	16,815	3,953

Notes: All models are estimated using logistic regressions and the reported coefficients are the average marginal effects. McFadden's $R^2 = 1 - ln(L_M)/ln(L_0)$ with L_M, the likelihood of the estimated model and L_0, the likelihood of the model without predictors. Robust standard errors in parentheses. ***, **, and * denote significance at the 1, 5, and 10 percent levels, respectively.

Source: Author's elaboration based on SAP (2011-16), HSES (2012-13), MICS (2006), LSMS (2012), EU-SILC (2013-15) and IAB-BAMF-SOEP Refugee Sample (2017).

Table A7.4: Selection on education of family-tied asylum seekers

Dependent variable: 1 if an individual has migrated, 0 otherwise
Benchmark group: Tertiary education

	Afghanistan			Iraq		Syria		
	(1)	(2)	(3)	(4)	(5)	(6)	(7)	(8)
Primary or less	−0.002	−0.002	−0.001	−0.002*	−0.003**	−0.022***	−0.022***	−0.022***
	(0.002)	(0.002)	(0.002)	(0.001)	(0.001)	(0.003)	(0.003)	(0.003)
Secondary	−0.002	−0.002	−0.001	−0.002**	−0.003**	−0.028***	−0.028***	−0.028***
	(0.002)	(0.002)	(0.002)	(0.001)	(0.001)	(0.003)	(0.003)	(0.003)
McFadden's R^2	0.017	0.049	0.068	0.008	0.078	0.025	0.122	0.130
Observations	50,499	50,499	50,499	80,882	80,882	52,680	52,680	52,680

Benchmark group: Secondary education or more

	Albania			Serbia		
	(10)	(11)	(12)	(13)	(14)	(15)
Primary or less	0.000	0.000	0.000	0.012***	0.012*	0.047***
	(0.000)	(0.000)	(0.000)	(0.004)	(0.007)	(0.017)
McFadden's R^2	0.026	0.069	0.066	0.368	0.369	0.445
Observations	14,836	14,836	14,836	33,406	33,406	8,369

Notes: All models are estimated using logistic regressions and the reported coefficients are the average marginal effects. McFadden's $R^2 = 1 - ln(L_M)/ln(L_0)$ with L_M, the likelihood of the estimated model and L_0, the likelihood of the model without predictors. Robust standard errors in parentheses. ***, **, and * denote significance at the 1, 5, and 10 percent levels, respectively.
Source: Author's elaboration based on SAP (2011-16), HSES (2012-13), MICS (2006), LSMS (2012), EU-SILC (2013-15) and IAB-BAMF-SOEP Refugee Sample (2017).
